# The genome sequence of the Orange-tailed Mining Bee, *Andrena haemorrhoa *(Fabricius, 1781)

**DOI:** 10.12688/wellcomeopenres.19982.1

**Published:** 2023-09-08

**Authors:** Liam M. Crowley, University of Oxford and Wytham Woods Genome Acquisition Lab, Darwin Tree of Life Barcoding collective, Wellcome Sanger Institute Tree of Life programme, Wellcome Sanger Institute Scientific Operations: DNA Pipelines collective, Tree of Life Core Informatics collective, Darwin Tree of Life Consortium

**Affiliations:** 1 University of Oxford, Oxford, England, UK

**Keywords:** Andrena haemorrhoa, Orange-tailed Mining Bee, genome sequence, chromosomal, Hymenoptera

## Abstract

We present a genome assembly from an individual female *Andrena haemorrhoa* (the Orange-tailed Mining Bee; Arthropoda; Insecta; Hymenoptera; Andrenidae). The genome sequence is 330.7 megabases in span. Most of the assembly is scaffolded into 7 chromosomal pseudomolecules. The mitochondrial genome has also been assembled and is 16.46 kilobases in length. Gene annotation of this assembly on Ensembl identified 10,908 protein coding genes.

## Species taxonomy

Eukaryota; Metazoa; Eumetazoa; Bilateria; Protostomia; Ecdysozoa; Panarthropoda; Arthropoda; Mandibulata; Pancrustacea; Hexapoda; Insecta; Dicondylia; Pterygota; Neoptera; Endopterygota; Hymenoptera; Apocrita; Aculeata; Apoidea; Anthophila; Andrenidae; Andreninae; *Andrena*; *Taeniandrena*; *Andrena haemorrhoa* (Fabricius, 1781) (NCBI:txid444401).

## Background

The Orange-tailed Mining Bee, *Andrena haemorrhoa*, is a widespread and locally common species of mining bee in the UK. It is widely distributed across all but the most southerly regions of Europe and can be one of the most abundant bee species where it occurs ( Banaszak-Cibicka & Żmihorski, 2012). It occurs across a wide variety of habitats and is only absent from the most extreme environments in its range. It is a medium-sized (7–10 mm forewing length) mining bee and both sexes are distinctive. Females have a neat pile of short, rich red hairs across the dorsal thorax, with white hairs on the face and sides of the thorax. The abdomen is sparsely haired, with a tuft of red hairs on the tip that give rise to the common name. The hairs of the males are more brownish-red with a reddish cluster of hairs on the tip of the abdomen. The hind tibiae and tarsi are orange, and males have a distinctive dark brown spot in the middle of the tibiae.

It is an early, univoltine *Andrena* species, with a flight period from March into July, leading to the alternative common name: the Early Mining Bee. Males typically emerge slightly earlier than females and may congregate around shrubs. After mating, females excavate nests with a preference for light soils, south facing banks, short swards and the margins of paths and tracks ( Falk & Lewington, 2019). Nesting often occurs in dispersed aggregations in suitable areas. It is widely polylectic, visiting a wide range of spring-flowering plants, especially Rosaceae including hawthorn ( *Crataegus monogyna*) ( Wood & Roberts, 2017), and may be an important pollinator of crops such as apple ( Kendall & Solomon, 1973).

The complete genome sequence for this species will facilitate studies into the evolution of mining bees, conservation of important pollinator species, reproductive evolution and foraging behaviour.

## Genome sequence report

**Figure 1.**
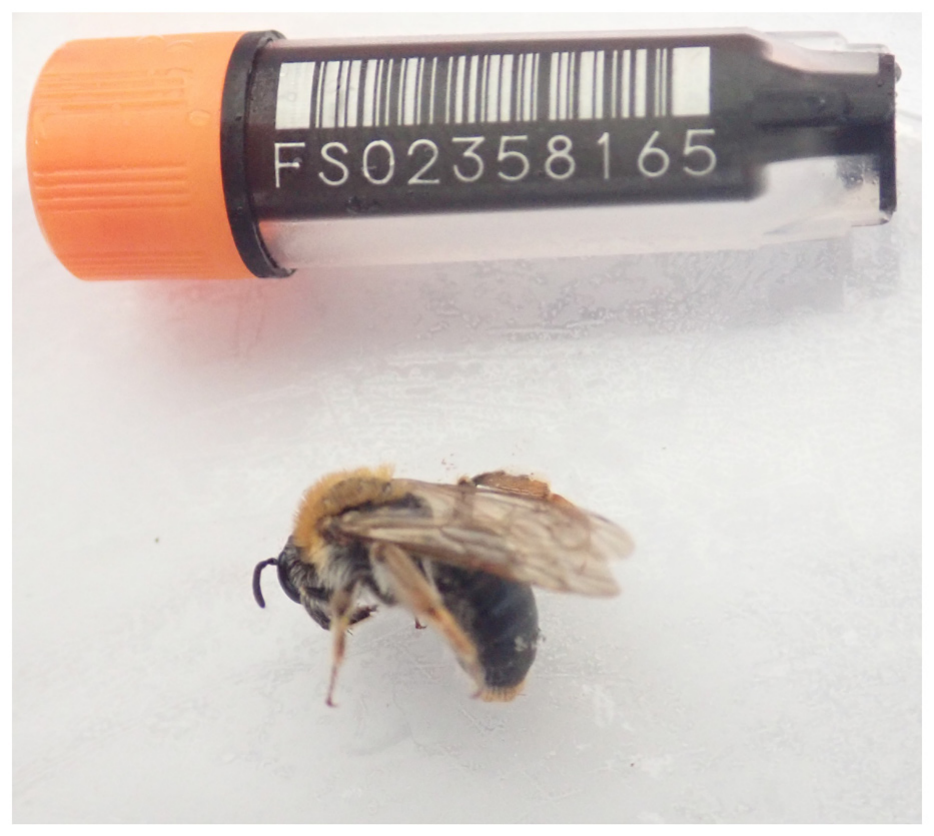
Photograph of the *Andrena haemorrhoa* (iyAndHaem1) specimen used for genome sequencing.

The genome was sequenced from one female *Andrena haemorrhoa* ( Figure 1) collected from Wytham Woods, Oxfordshire, UK (51.77, –1.34). A total of 77-fold coverage in Pacific Biosciences single-molecule HiFi long reads and 91-fold coverage in 10X Genomics read clouds were generated. Primary assembly contigs were scaffolded with chromosome conformation Hi-C data. Manual assembly curation corrected 27 missing joins or mis-joins and removed 1 haplotypic duplication, reducing the assembly length by 0.24% and the scaffold number by 4.74%, and increasing the scaffold N50 122.57%.

**Figure 2.**
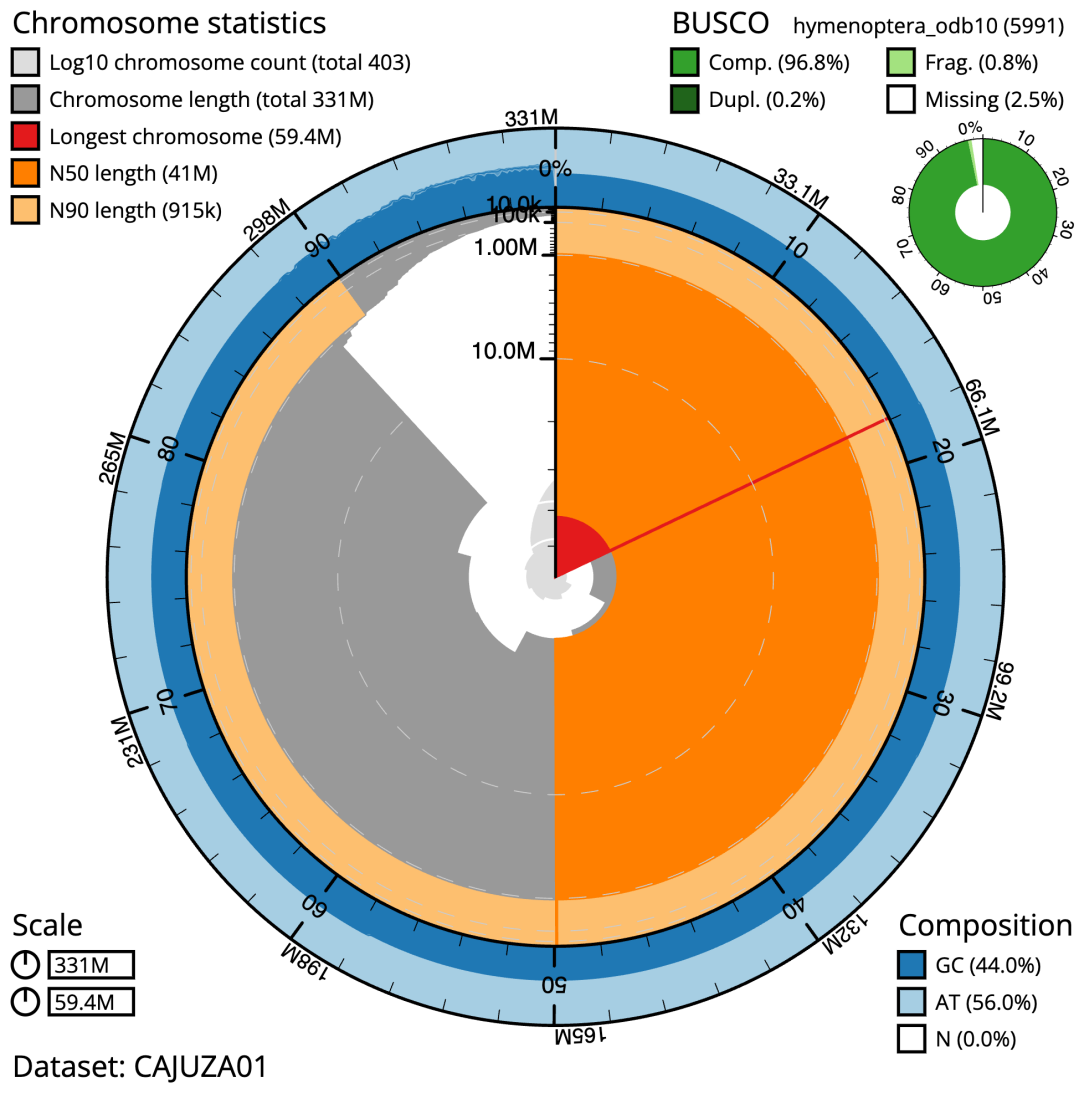
Genome assembly of *Andrena haemorrhoa*, iyAndHaem1.1: metrics. The BlobToolKit Snailplot shows N50 metrics and BUSCO gene completeness. The main plot is divided into 1,000 size-ordered bins around the circumference with each bin representing 0.1% of the 330,687,150 bp assembly. The distribution of scaffold lengths is shown in dark grey with the plot radius scaled to the longest scaffold present in the assembly (59,446,405 bp, shown in red). Orange and pale-orange arcs show the N50 and N90 scaffold lengths (41,012,438 and 915,300 bp), respectively. The pale grey spiral shows the cumulative scaffold count on a log scale with white scale lines showing successive orders of magnitude. The blue and pale-blue area around the outside of the plot shows the distribution of GC, AT and N percentages in the same bins as the inner plot. A summary of complete, fragmented, duplicated and missing BUSCO genes in the hymenoptera_odb10 set is shown in the top right. An interactive version of this figure is available at https://blobtoolkit.genomehubs.org/view/iyAndHaem1.1/dataset/CAJUZA01/snail.

The final assembly has a total length of 330.7 Mb in 402 sequence scaffolds with a scaffold N50 of 41.0 Mb ( Table 1). Most (88.08%) of the assembly sequence was assigned to 7 chromosomal-level scaffolds. Chromosome-scale scaffolds confirmed by the Hi-C data are named in order of size ( Figure 2– Figure 5; Table 2). The specimen is a diploid female. While not fully phased, the assembly deposited is of one haplotype. Contigs corresponding to the second haplotype have also been deposited. The mitochondrial genome was also assembled and can be found as a contig within the multifasta file of the genome submission.

**Table 1.** Genome data for *Andrena haemorrhoa*, iyAndHaem1.1.

Project accession data		
Assembly identifier	iyAndHaem1.1	
Species	*Andrena haemorrhoa*	
Specimen	iyAndHaem1	
NCBI taxonomy ID	444401	
BioProject	PRJEB45180	
BioSample ID	SAMEA7520535	
Isolate information	iyAndHaem1, female: abdomen (DNA sequencing), head and thorax (Hi-C sequencing) iyAndHaem3, male: abdomen (RNA sequencing)	
Assembly metrics *		*Benchmark*
Consensus quality (QV)	51.3	*≥ 50*
*k*-mer completeness	99.97%	*≥ 95%*
BUSCO **	C:96.8%[S:96.5%,D:0.2%], F:0.8%,M:2.5%,n:5,991	*C ≥ 95%*
Percentage of assembly mapped to chromosomes	88.08%	*≥ 95%*
Sex chromosomes	-	*localised homologous pairs*
Organelles	Mitochondrial genome assembled	*complete single alleles*
Raw data accessions		
PacificBiosciences SEQUEL II	ERR6412038	
10X Genomics Illumina	ERR6054883, ERR6054886, ERR6054888, ERR6054884, ERR6054885, ERR6054881, ERR6054882, ERR6054887	
Hi-C Illumina	ERR6054880	
PolyA RNA-Seq Illumina	ERR9434982	
Genome assembly		
Assembly accession	GCA_910592295.1	
*Accession of alternate haplotype*	GCA_910592125.1	
Span (Mb)	330.7	
Number of contigs	439	
Contig N50 length (Mb)	13.2	
Number of scaffolds	402	
Scaffold N50 length (Mb)	41.0	
Longest scaffold (Mb)	59.5	
Genome annotation		
Number of protein-coding genes	10,908	
Number of non-coding genes	4,411	
Number of gene transcripts	23,723	

* Assembly metric benchmarks are adapted from column VGP-2020 of “Table 1: Proposed standards and metrics for defining genome assembly quality” from ( Rhie *et al.*, 2021). ** BUSCO scores based on the hymenoptera_odb10 BUSCO set using v5.3.2. C = complete [S = single copy, D = duplicated], F = fragmented, M = missing, n = number of orthologues in comparison. A full set of BUSCO scores is available at https://blobtoolkit.genomehubs.org/view/iyAndHaem1.1/dataset/CAJUZA01/busco.

**Figure 3.**
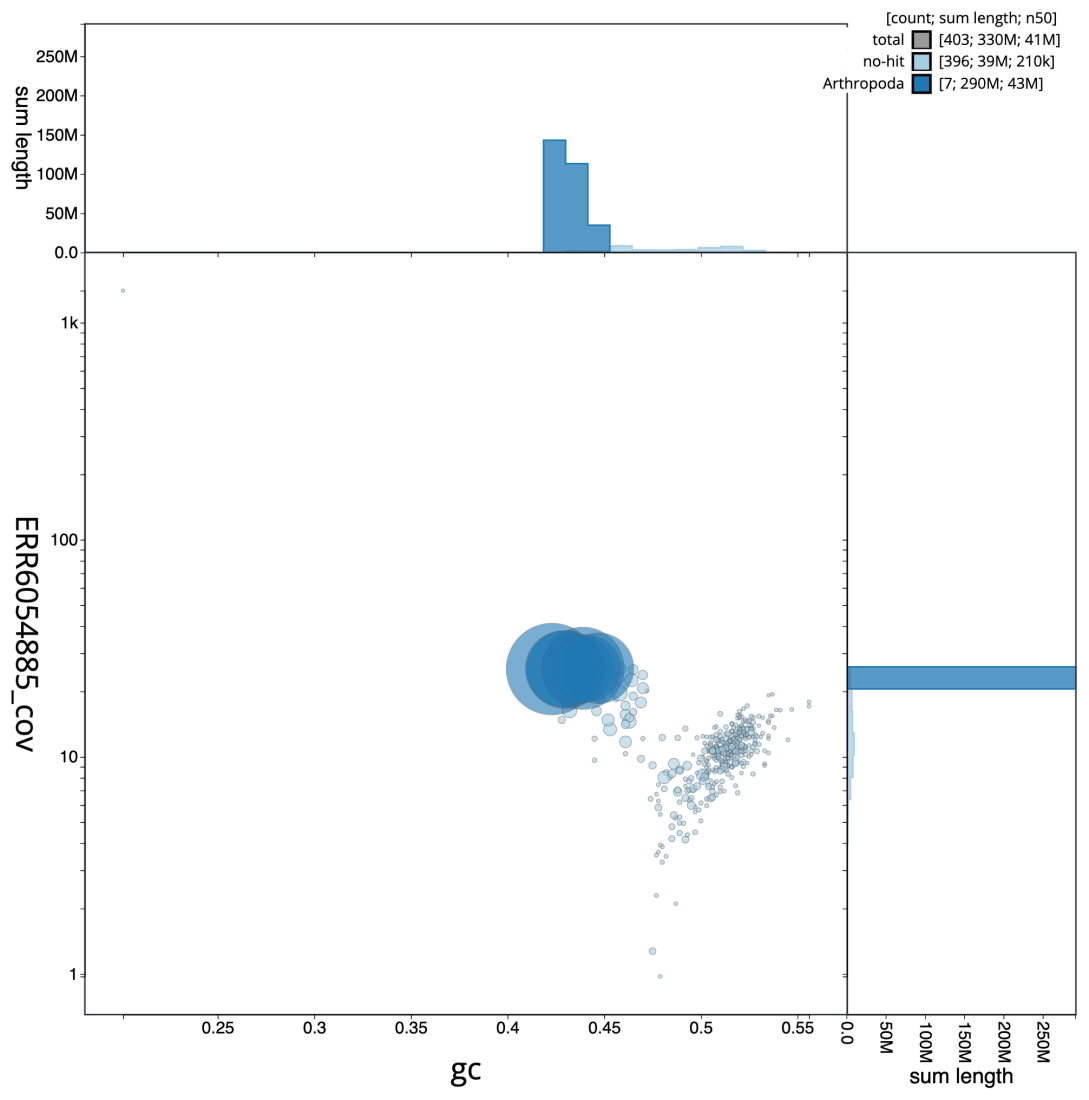
Genome assembly of *Andrena haemorrhoa*, iyAndHaem1.1: BlobToolKit GC-coverage plot. Scaffolds are coloured by phylum. Circles are sized in proportion to scaffold length. Histograms show the distribution of scaffold length sum along each axis. An interactive version of this figure is available at https://blobtoolkit.genomehubs.org/view/iyAndHaem1.1/dataset/CAJUZA01/blob.

**Figure 4.**
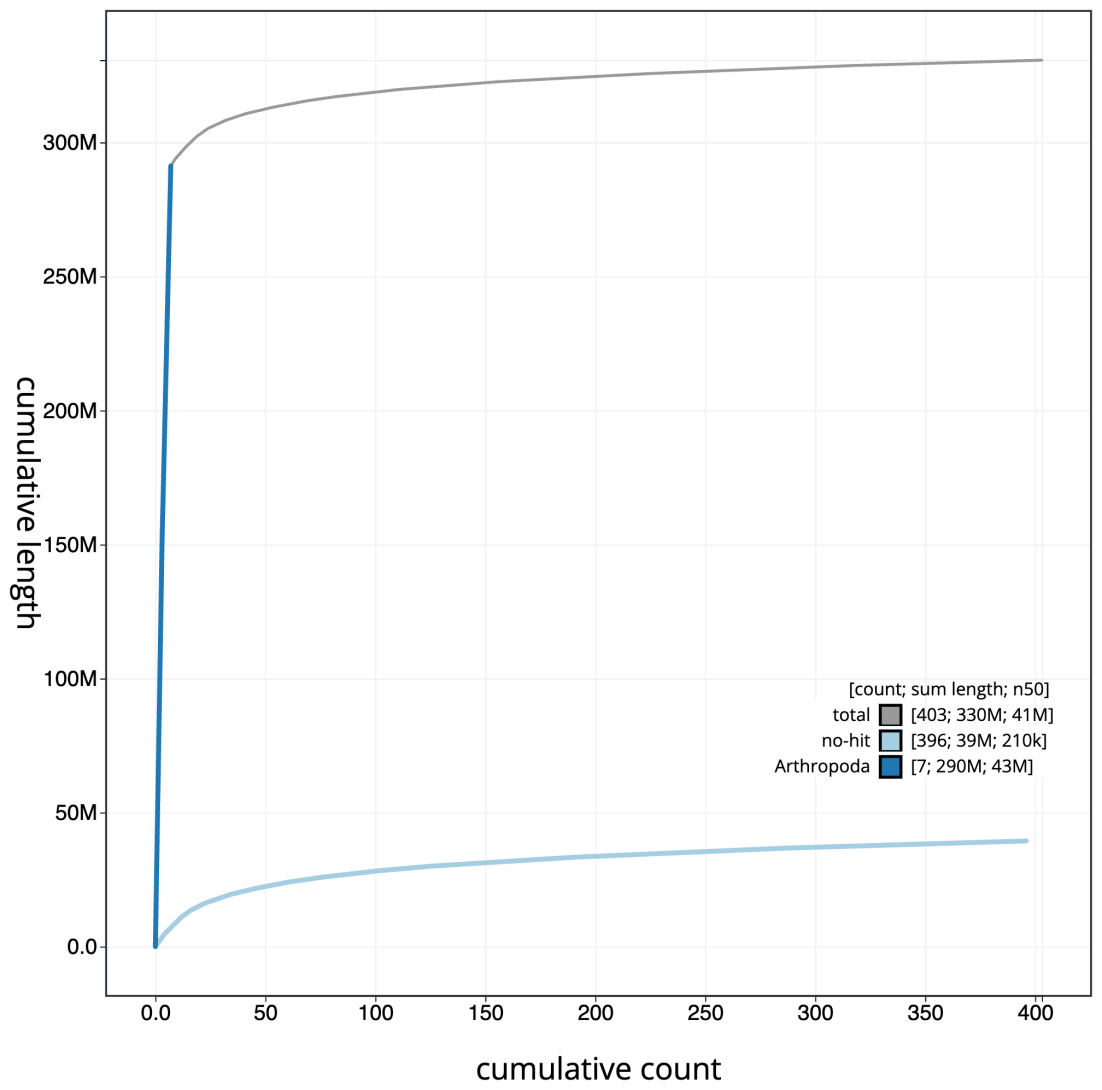
Genome assembly of *Andrena haemorrhoa*, iyAndHaem1.1: BlobToolKit cumulative sequence plot. The grey line shows cumulative length for all scaffolds. Coloured lines show cumulative lengths of scaffolds assigned to each phylum using the buscogenes taxrule. An interactive version of this figure is available at https://blobtoolkit.genomehubs.org/view/iyAndHaem1.1/dataset/CAJUZA01/cumulative.

**Figure 5.**
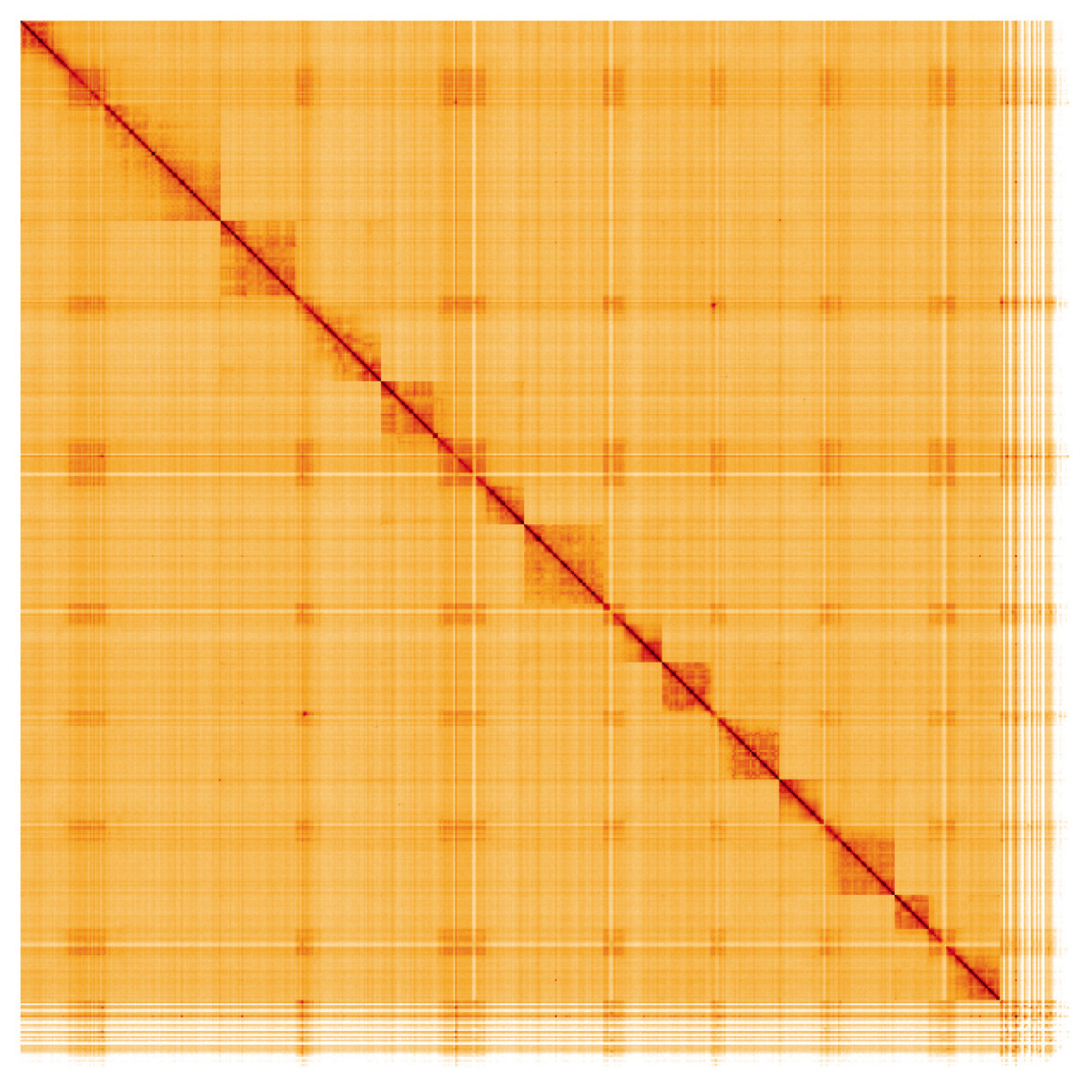
Genome assembly of *Andrena haemorrhoa*, iyAndHaem1.1: Hi-C contact map of the iyAndHaem1.1 assembly, visualised using HiGlass. Chromosomes are shown in order of size from left to right and top to bottom. An interactive version of this figure may be viewed at https://genome-note-higlass.tol.sanger.ac.uk/l/?d=JrLYTlFyTR2idPajNfYFDg.

**Table 2.** Chromosomal pseudomolecules in the genome assembly of *Andrena haemorrhoa*, iyAndHaem1.

INSDC accession	Chromosome	Length (Mb)	GC%
OU342940.1	1	59.45	42.5
OU342941.1	2	47.58	44.0
OU342942.1	3	42.63	43.0
OU342943.1	4	41.01	43.0
OU342944.1	5	34.84	44.5
OU342945.1	6	34.54	43.5
OU342946.1	7	31.21	44.0
OU342947.1	MT	0.02	20.5

The estimated Quality Value (QV) of the final assembly is 51.3 with *k*-mer completeness of 99.97%, and the assembly has a BUSCO v5.3.2 completeness of 96.8% (single = 96.5%, duplicated = 0.2%), using the hymenoptera_odb10 reference set ( *n* = 5,991).

Metadata for specimens, spectral estimates, sequencing runs, contaminants and pre-curation assembly statistics can be found at https://links.tol.sanger.ac.uk/species/444401.

## Genome annotation report

The *Andrena haemorrhoa* genome assembly (GCA_910592295.1) was annotated using the Ensembl rapid annotation pipeline ( Table 1; https://rapid.ensembl.org/Andrena_haemorrhoa_GCA_910592295.1/Info/Index). The resulting annotation includes 23,723 transcribed mRNAs from 10,908 protein-coding and 4,411 non-coding genes.

## Methods

### Sample acquisition and nucleic acid extraction

The specimen used for DNA and Hi-C sequencing was a female *Andrena haemorrhoa* (specimen ID Ox000414, ToLID iyAndHaem1), which was netted in Wytham Woods, Oxfordshire (biological vice-county Berkshire), UK (latitude 51.77, longitude –1.34) on 2020-05-22. The specimen used for RNA sequencing was a male *Andrena haemorrhoa* (specimen ID Ox001075, ToLID iyAndHaem3), collected from the same location on 2021-03-29. Liam Crowley (University of Oxford) collected and identified both specimens. The specimens were snap-frozen on dry ice.

DNA was extracted at the *Andrena haemorrhoa* Tree of Life laboratory, Wellcome Sanger Institute (WSI). The iyAndHaem1 sample was weighed and dissected on dry ice with head and thorax tissue set aside for Hi-C sequencing. Abdomen tissue was disrupted using a Nippi Powermasher fitted with a BioMasher pestle. High molecular weight (HMW) DNA was extracted using the Qiagen MagAttract HMW DNA extraction kit. Low molecular weight DNA was removed from a 20 ng aliquot of extracted DNA using the 0.8X AMpure XP purification kit prior to 10X Chromium sequencing; a minimum of 50 ng DNA was submitted for 10X sequencing. HMW DNA was sheared into an average fragment size of 12–20 kb in a Megaruptor 3 system with speed setting 30. Sheared DNA was purified by solid-phase reversible immobilisation using AMPure PB beads with a 1.8X ratio of beads to sample to remove the shorter fragments and concentrate the DNA sample. The concentration of the sheared and purified DNA was assessed using a Nanodrop spectrophotometer and Qubit Fluorometer and Qubit dsDNA High Sensitivity Assay kit. Fragment size distribution was evaluated by running the sample on the FemtoPulse system.

RNA was extracted from abdomen tissue of iyAndHaem3 in the Tree of Life Laboratory at the WSI using TRIzol, according to the manufacturer’s instructions. RNA was then eluted in 50 μl RNAse-free water and its concentration assessed using a Nanodrop spectrophotometer and Qubit Fluorometer using the Qubit RNA Broad-Range (BR) Assay kit. Analysis of the integrity of the RNA was done using Agilent RNA 6000 Pico Kit and Eukaryotic Total RNA assay.

### Sequencing

Pacific Biosciences HiFi circular consensus and 10X Genomics read cloud DNA sequencing libraries were constructed according to the manufacturers’ instructions. Poly(A) RNA-Seq libraries were constructed using the NEB Ultra II RNA Library Prep kit. DNA and RNA sequencing was performed by the Scientific Operations core at the WSI on Pacific Biosciences SEQUEL II (HiFi), Illumina HiSeq 4000 (RNA-Seq) and HiSeq X Ten (10X) instruments. Hi-C data were also generated from remaining head and thorax tissue of iyAndHaem1 using the Arima2 kit and sequenced on the HiSeq X Ten instrument.

### Genome assembly, curation and evaluation

Assembly was carried out with Hifiasm ( Cheng *et al.*, 2021) and haplotypic duplication was identified and removed with purge_dups ( Guan *et al.*, 2020). One round of polishing was performed by aligning 10X Genomics read data to the assembly with Long Ranger ALIGN, calling variants with FreeBayes ( Garrison & Marth, 2012). The assembly was then scaffolded with Hi-C data ( Rao *et al.*, 2014) using SALSA2 ( Ghurye *et al.*, 2019). The assembly was checked for contamination and corrected using the gEVAL system ( Chow *et al.*, 2016) as described previously ( Howe *et al.*, 2021). Manual curation was performed using gEVAL, HiGlass ( Kerpedjiev *et al.*, 2018) and Pretext ( Harry, 2022). The mitochondrial genome was assembled using MitoHiFi ( Uliano-Silva *et al.*, 2023), which runs MitoFinder ( Allio *et al.*, 2020) or MITOS ( Bernt *et al.*, 2013) and uses these annotations to select the final mitochondrial contig and to ensure the general quality of the sequence.

A Hi-C map for the final assembly was produced using bwa-mem2 ( Vasimuddin *et al.*, 2019) in the Cooler file format ( Abdennur & Mirny, 2020). To assess the assembly metrics, the *k*-mer completeness and QV consensus quality values were calculated in Merqury ( Rhie *et al.*, 2020). This work was done using Nextflow ( Di Tommaso *et al.*, 2017) DSL2 pipelines “sanger-tol/readmapping” ( Surana *et al.*, 2023a) and “sanger-tol/genomenote” ( Surana *et al.*, 2023b). The genome was analysed within the BlobToolKit environment ( Challis *et al.*, 2020) and BUSCO scores ( Manni *et al.*, 2021; Simão *et al.*, 2015) were calculated.

Table 3 contains a list of relevant software tool versions and sources.

**Table 3.** Software tools: versions and sources.

Software tool	Version	Source
BlobToolKit	3.5.0	https://github.com/blobtoolkit/blobtoolkit
BUSCO	5.3.2	https://gitlab.com/ezlab/busco
FreeBayes	1.3.1-17- gaa2ace8	https://github.com/freebayes/freebayes
gEVAL	N/A	https://geval.org.uk/
Hifiasm	0.12	https://github.com/chhylp123/hifiasm
HiGlass	1.11.6	https://github.com/higlass/higlass
Long Ranger ALIGN	2.2.2	https://support.10xgenomics.com/genome- exome/software/pipelines/latest/ advanced/other-pipelines
Merqury	MerquryFK	https://github.com/thegenemyers/MERQURY.FK
MitoHiFi	2	https://github.com/marcelauliano/MitoHiFi
PretextView	0.2	https://github.com/wtsi-hpag/PretextView
purge_dups	1.2.3	https://github.com/dfguan/purge_dups
SALSA	2.2	https://github.com/salsa-rs/salsa
sanger-tol/ genomenote	v1.0	https://github.com/sanger-tol/genomenote
sanger-tol/ readmapping	1.1.0	https://github.com/sanger-tol/readmapping/ tree/1.1.0

### Genome annotation

The Ensembl gene annotation system ( Aken *et al.*, 2016) was used to generate annotation for the *Andrena haemorrhoa* assembly (GCA_910592295.1). Annotation was created primarily through alignment of transcriptomic data to the genome, with gap filling via protein-to-genome alignments of a select set of proteins from UniProt ( UniProt Consortium, 2019).

### Wellcome Sanger Institute – Legal and Governance

The materials that have contributed to this genome note have been supplied by a Darwin Tree of Life Partner. The submission of materials by a Darwin Tree of Life Partner is subject to the **‘Darwin Tree of Life Project Sampling Code of Practice’**, which can be found in full on the Darwin Tree of Life website here. By agreeing with and signing up to the Sampling Code of Practice, the Darwin Tree of Life Partner agrees they will meet the legal and ethical requirements and standards set out within this document in respect of all samples acquired for, and supplied to, the Darwin Tree of Life Project. 

Further, the Wellcome Sanger Institute employs a process whereby due diligence is carried out proportionate to the nature of the materials themselves, and the circumstances under which they have been/are to be collected and provided for use. The purpose of this is to address and mitigate any potential legal and/or ethical implications of receipt and use of the materials as part of the research project, and to ensure that in doing so we align with best practice wherever possible. The overarching areas of consideration are:

• Ethical review of provenance and sourcing of the material

• Legality of collection, transfer and use (national and international) 

Each transfer of samples is further undertaken according to a Research Collaboration Agreement or Material Transfer Agreement entered into by the Darwin Tree of Life Partner, Genome Research Limited (operating as the Wellcome Sanger Institute), and in some circumstances other Darwin Tree of Life collaborators.

## Data Availability

European Nucleotide Archive: *Andrena haemorrhoa* (red-tailed mining bee). Accession number PRJEB45180; https://identifiers.org/ena.embl/PRJEB45180. ( Wellcome Sanger Institute, 2021) The genome sequence is released openly for reuse. The *Andrena haemorrhoa* genome sequencing initiative is part of the Darwin Tree of Life (DToL) project. All raw sequence data and the assembly have been deposited in INSDC databases. Raw data and assembly accession identifiers are reported in Table 1.

## Author information

Members of the University of Oxford and Wytham Woods Genome Acquisition Lab are listed here: https://doi.org/10.5281/zenodo.4789928.

Members of the Darwin Tree of Life Barcoding collective are listed here: https://doi.org/10.5281/zenodo.4893703.

Members of the Wellcome Sanger Institute Tree of Life programme are listed here: https://doi.org/10.5281/zenodo.4783585.

Members of Wellcome Sanger Institute Scientific Operations: DNA Pipelines collective are listed here: https://doi.org/10.5281/zenodo.4790455.

Members of the Tree of Life Core Informatics collective are listed here: https://doi.org/10.5281/zenodo.5013541.

Members of the Darwin Tree of Life Consortium are listed here: https://doi.org/10.5281/zenodo.4783558.
