# Supplementary material for: The genome sequence of the Orange-tailed Mining Bee, Andrena haemorrhoa (Fabricius, 1781)
Source: Wellcome Open Res. 2023 Sep 8;8:396. [Version 1] doi: 10.12688/wellcomeopenres.19982.1 (PMC11134145; doi:10.12688/wellcomeopenres.19982.1)
Supplement: Peer review history

# Reviewer report: Reviewer response for version 1

**DOI:** 10.21956/wellcomeopenres.22128.r72329

**Published:** 2024-05-28

**Authors:** James B. Whitfield

**Affiliations:** 1 Department of Entomology, University of Illinois at Urbana-Champaign, Urbana, IL, USA

This report provides the genome sequence and associated details for the orange-tailed mining bee, *Andrena haemorrhoa*, a common familiar early spring solitary bee.  The paper is very cleanly and clearly prepared and presented, and adds significantly to our knowledge of wild bee genomics. This work is of relevance due to the increasing recognition of the value of native pollinators, and the need for greater understanding of their biology and ecophysiological requirements.

 The manuscript presents the usual comparative statistics concerning genome size, organization and content, and the assembly quality seems to be reasonably high, allowing useful comparisons with other available bee genomes. Especially relevant gene families to compare might include olfactory and gustatory receptors, detoxification enzymes and heat tolerance factors.

 There did not, however, seem to be any direct comparisons to other published *Andrena* genomes (which seem to show considerable similarity); perhaps the one I am aware of was published too recently for the authors to have seen?  This omission does not necessarily detract from the value of the paper in presenting the new genome, but if it is not too late, it would be interesting for the authors to compare to:

 (Zhao et al. 2023) [ Ref1 ]. A chromosome-level genome assembly and evolution analysis of *Andrena camellia* ( *Hymennoptera: Andrenidae*). Genome Biology and Evolution 15 (5):evad080

Are sufficient details of methods and materials provided to allow replication by others?

Yes

Is the rationale for creating the dataset(s) clearly described?

Yes

Are the datasets clearly presented in a useable and accessible format?

Yes

Are the protocols appropriate and is the work technically sound?

Yes

Reviewer Expertise:

Phylogenetics, ecology and genomics of parasitoid wasps, especially Braconidae

I confirm that I have read this submission and believe that I have an appropriate level of expertise to confirm that it is of an acceptable scientific standard.

## References

[1] : A Chromosome-level Genome Assembly and Evolution Analysis of Andrena camellia (Hymenoptera: Andrenidae). *Genome Biol Evol* .2023;15(5) : 10.1093/gbe/evad080 10.1093/gbe/evad080 37170910 PMC10210616

# Reviewer report: Reviewer response for version 1

**DOI:** 10.21956/wellcomeopenres.22128.r75008

**Published:** 2024-05-28

**Authors:** Thiago Mafra Batista

**Affiliations:** 1 Centro de Formacao em Ciencias Ambientais, Universidade Federal do Sul da Bahia, Porto Seguro, State of Bahia, Brazil

This study describes the genome of the Andrena haemorrhoa bee, sequenced with 77-fold coverage using PacBio HiFi and 91-fold coverage using 10X Genomics. The assembly boasts an N50 of 41 Mb. The contigs were scaffolded using Hi-C into 7 chromosomal pseudomolecules, accounting for 88.08% of the genome. The genome completeness is 96.8% (BUSCO Hymenoptera_odb10 n= 5,991). The methods are clear, detailed, and reproducible, and metadata is available.

 Strengths:

**High Coverage** **:** The 77-fold coverage with PacBio HiFi and 91-fold coverage with 10X Genomics significantly enhance the accuracy and reliability of the genome assembly.

**Quality Assembly**: An N50 of 41 Mb indicates a high-quality assembly, suggesting long and contiguous contigs.

**Use of Advanced Technologies:** The effective combination of cutting-edge sequencing technologies such as PacBio HiFi, 10X Genomics, and Hi-C showcases a robust approach to achieving a high-quality genome assembly.

**Chromosomal Pseudomolecule Organization**: The assembly into 7 chromosomal pseudomolecules, covering 88.08% of the genome, provides a useful framework for future structural and functional genomic studies.

**High Genome Completeness**: The 96.8% completeness (BUSCO) demonstrates that the majority of conserved genes are present, essential for the functional integrity of the sequenced genome.

**Transparency and Reproducibility:** Detailed methods and the availability of metadata ensure that other researchers can replicate the study, promoting scientific transparency.

 Weaknesses:

**Partial Genome Coverage:** While 88.08% of the genome is covered, 11.92% remains unrepresented in the chromosomal pseudomolecules. The study could benefit from a discussion on the missing genomic content and its potential implications.

 Suggestions for Improvement:

 Additional Information in Table 2: Include a column in Table 2 with information on the percentage of the genome each pseudomolecule represents, cumulatively totaling 88.08%.

Are sufficient details of methods and materials provided to allow replication by others?

Yes

Is the rationale for creating the dataset(s) clearly described?

Yes

Are the datasets clearly presented in a useable and accessible format?

Yes

Are the protocols appropriate and is the work technically sound?

Yes

Reviewer Expertise:

Genomics

I confirm that I have read this submission and believe that I have an appropriate level of expertise to confirm that it is of an acceptable scientific standard.

# Reviewer report: Reviewer response for version 1

**DOI:** 10.21956/wellcomeopenres.22128.r66789

**Published:** 2023-09-26

**Authors:** Trevor Sless

**Affiliations:** 1 Biology, York University, Toronto, Ontario, Canada

Summary:

 The submitted paper presents the complete genome sequence of the mining bee *Andrena haemorrhoa*. This marks the first representative of the genus *Andrena* (as well as the family Andrenidae) to have a genome assembly released, alongside several other recently sequenced *Andrena* species from the Wellcome Sanger Institute and Chongqing Normal University.

 The combined use of 10X Genomics, HiFi, and Hi-C sequencing technologies is in keeping with the latest standards in the field of genomics and ensures a high-quality genome, as borne out by the resulting assembly statistics described in the paper. The annotation methodology is similarly robust.

 All assembly and raw read datasets have been made publicly available as indicated in the data availability section and will be a valuable resource to future studies into the genetics and genomics of *Andrena haemorrhoa* and related species. In summary, I recommend that the paper be accepted for indexing in Wellcome Open Research, but provide a few small suggestions as detailed below.

Specific Suggestions:

 Background:

 “It is widely distributed across all but the most southerly regions of Europe…”

 Based on GBIF records, *Andrena haemorrhoa *is widespread throughout the Palearctic including as far east as Japan, while this text seems to suggest it is only found in Europe; please amend.

 Methods:

 “Low molecular weight DNA was removed from a 20 ng aliquot of extracted DNA using the 0.8X AMpure XP purification kit prior to 10X Chromium sequencing; a minimum of 50 ng DNA was submitted for 10X sequencing.”

 Was the purification kit also used on the HMW DNA sent for HiFi sequencing, or only the 10X Chromium material?

 Table 1:

 The “Percentage of assembly mapped to chromosomes” metric stands out slightly as the only one which does not reach the provided benchmark value. Though I realize the scope of this paper does not leave much space for discussion, are there any possible explanations for this (e.g., perhaps microchromosomes/B chromosomes, or methodological factors?)

 Figure 3:

 I feel that this figure would benefit from rescaling the axes to make better use of the available space. Could the outlier point (which I assume represents the mitogenome) be removed or otherwise included as an inset to allow this?

Are sufficient details of methods and materials provided to allow replication by others?

Yes

Is the rationale for creating the dataset(s) clearly described?

Yes

Are the datasets clearly presented in a useable and accessible format?

Yes

Are the protocols appropriate and is the work technically sound?

Yes

Reviewer Expertise:

Bees, Evolution, Genomics, Phylogenetics

I confirm that I have read this submission and believe that I have an appropriate level of expertise to confirm that it is of an acceptable scientific standard.
